# Supplementary material for: Risk Assessment of Fenpropathrin: Cause Hepatotoxicity and Nephrotoxicity in Common Carp (Cyprinus carpio L.)
Source: Int J Mol Sci. 2024 Sep 11;25(18):9822. doi: 10.3390/ijms25189822 (PMC11432585; doi:10.3390/ijms25189822)
Supplement: Supplementary file 1 [file ijms-25-09822-s001.zip › ijms-3187658-supplementary.pdf]

International Journal of
Molecular Sciences

MDPI

Supplementary materials

Risk assessment of fenpropathrin: cause hepatotoxicity and nephrotoxicity in common carp (*Cyprinus carpio* L.)

Gongming Zhu [1,2,3†], Zhihui Liu [1,2†], Hao Wang [1,2], Shaoyu Mou [1,2], Yuanyuan Li [1,2], Junguo Ma [1,2,3*] and Xiaoyu Li [2]

[1] State Key Laboratory of Antiviral Drugs, College of Life Science, Henan Normal University, Xinxiang 453007, China; 2018208@htu.edu.cn (G.Z.); liuzh2958@163.com (Z.L.); 15516748299@163.com (H.W.); 18439513168@163.com (S.M.); 15993030397@126.com (Y.L.); 041035@htu.edu.cn (X.L.)
[2] Henan International Joint Laboratory of Aquatic Toxicology and Health Protection, Henan Normal University, Xinxiang 453007, China;
[3] Pingyuan Laboratory, Xinxiang 453007, China
* Correspondence: 2016023@htu.edu.cn
† These authors contributed equally to this work.

1. Supplementary Methods

Methods S1. Biochemical indicators detection

The blood samples were collected from the common carp caudal vein, let stand for 2 hours in the lab, stored overnight at 4 °C, and subsequently subjected to centrifugation (4 °C, 4000 g for 15 min) to obtain serum for biochemical analyses, which was then stored at −20 °C.

The samples of the common carp liver and kidney tissues were homogenized in 0.9% ice-cold saline solution at a ratio of 1:9 (w/v). Subsequently, the homogenate was centrifuged at 10, 000 rpm at 4 °C for 10 min, followed by collection of the supernatant for biochemical analyses. The concentration of the protein was measured by Bradford protein concentration assay kit (# P006, Beyotime, Shanghai , China).

The ALT, AST, SOD, ALP, and CAT activities, and the contents of GLU, TG, T-CHO, ALB and MDA were measured by biochemical kits.

The serum ALT and AST activities were measured according to the kit protocol (Nanjing Jiancheng, Nanjing, China). Set the assay and control empty space, added the matrix solution preheated at 37 °C, and then the sample to be tested was added for a series of reactions. Finally, the microplate reader (SpectraMax 190, USA) measured the OD value of each well at 510 nm, and the absolute OD value was substituted into the standard curve to obtain the corresponding ALT or AST activities.

The activities of SOD were assessed by the kit protocol (Nanjing Jiancheng, Nanjing, China). The samples to be tested and working solutions with different effects were added to the wells following the provided instructions. Subsequently, the mixture was incubated at 37 ℃ for 20 min and read using a microplate reader at a wavelength of 450 nm, and calculated the SOD activities according to the formula.

The decomposition of ALP yielded free phenol, which was oxidized by potassium ferricyanide with 4-amino-antipyrin in alkaline solution to generate red Quinone derivatives, ALP enzyme activity can be determined according to the shade of red. Different working solutions and samples to be tested were added, mixed thoroughly, and bathed at 37 ℃ for 15 min and carried out color reaction. Finally, a microplate reader detected the value of absorbance at 520 nm in each well.

In addition, the catalytic decomposition of H2O2 by CAT was rapidly inhibited upon the introduction of ammonium molybdate, leading to the formation of a light-yellow complex through reaction with the remaining H2O2. The activities of CAT were determined by monitoring the absorbance changes at 405 nm.

The contents of GLU, TG, T-CHO and ALB were measured following the kit protocol (Nanjing Jiancheng, Nanjing, China). Added 2.5 μL distilled water, 2.5 μL standard and 2.5 μL sample to blank, standard and sample Wells, respectively, and added 250 μL working solution to all Wells, mixed, and incubated at 37 ℃ for 10 min. Microplate reader measured the absorbance value of each well (GLU at 505 nm, TG and T-CHO at 510 nm, ALB at 628/630 nm).

MDA condensed with TBA leads to form red products exhibiting the most maximum absorption peak at 532 nm. The MDA contents were measured according to the method of TBA (Nanjing Jiancheng, Nanjing, China). Blank, standard, measuring and control tubes were set, and the working solution and samples were added follwong the kit's instructions. After mixing, boiled for 40 min, cooled to room temperature and centrifuged at 3500-4000 rpm for 15 min to make the precipitation complete. The supernatant was taken and the absorbance value was determined.

ELISA kits were used for determining the contents of ROS, TNF-α, and IL-1β, the experimental procedures were performed as follows.

The first step was dilute the standards to make a standard curve (Set ten Standard wells on the microplate, and 100 μL Standard were added to the first and second Wells, respectively, and then successively diluted to the corresponding concentration gradients). Secondly, the blank Wells and the sample Wells to be tested were set up, followed by incubation, washing, and addition of enzyme-labeled reagents and repeated incubation and washing. Finally, the color was developed at 37 ℃, followed by the quantification of absorbance value at a wavelength of 450 nm using a microplate reader (SpectraMax 190, USA).

Methods S2. Transcriptome analysis

Total RNA extraction, RNA integrity evaluation, libraries construction, and the transcriptome sequencing were performed as previously description [40]. The FPKM [61] and read counts values for each transcript (protein-coding) were calculated by bowtie2 [62] and eXpress [63]. DEGs were analysis by the DESeq method [64]. GO and KEGG analysis of DEGs were performed as previously established protocol [65]. Additionally, PPI networks were constructed throuth the STRING database, and visualized using Cytoscape software (v3.9.1).

Methods S3. Quantitative PCR (qPCR)

The tissues of common carp liver and kidney were collected to extract RNA by a RNAiso Plus Kit (Takara, Dalian, China). The purity, quantification and concentrations of total RNA were measured using the NanoDrop 2000 spectrophotometer. The RNA integrity of samples was assayed by 1% agarose formaldehyde gel electrophoresis with EB. Total RNA was converted into first-strand cDNA by a HiFi-Script cDNA Synthesis Kit (CoWin Biosciences, Beijing, China), and then, target genes were selected for confirmation by qPCR with an SYBR Green qPCR Mix (Monad, Shanghai, China). The *ef-1α* mRNA was employed as the inner reference gene [66]. The reaction programs were performed following the kit's protocols. The levels of target gene expression in carp liver and kidney were computed by the $2^{-\triangle\triangle Ct}$ method as Livak and Schmittgen [67] described. The qPCR was conducted in accordance with the MIQE guidelines [68].

References

40. Wang, W.; Mou, S.; Xiu, W.; Li, Y.; Liu, Z.; Feng, Y.; Ma, J.; Li, X. Fenpropathrin disrupted the gills of common carp (Cyprinus carpio L.) through oxidative stress, inflammatory responses, apoptosis, and transcriptional alterations. *Ecotoxicol. Environ. Safe*. **2024**, 271, 116007.
61. Kim, D.; Langmead, B.; Salzberg, S.L. HISAT: a fast spliced aligner with low memory requirements. *Nat. Methods.* **2015**, 12, 357-360.
62. Langmead, B.; Salzberg, S.L. Fast gapped-read alignment with Bowtie 2. *Nat. Methods.* **2012**, 9, 357-359.
63. Roberts, A.; Pachter, L. Streaming fragment assignment for real-time analysis of sequencing experiments. *Nat. Methods.* **2013**, 10, 71-73.
64. Anders, S.; Huber, W. Differential expression of RNA-Seq data at the gene level - the DESeq package. *Heidelb. Ger. Eur. Mol. Biol. Lab*. **2012**, 10, 1000.
65. Kanehisa, M.; Araki, M.; Goto, S.; Hattori, M.; Hirakawa, M.; Itoh, M.; Katayama, T.; Kawashima, S.; Okuda, S.; Tokimatsu, T.; Yamanishi, Y. KEGG for linking genomes to life and the environment. *Nucleic Acids Res.* **2008**, 36, 480-484.
66. Wang, X.Y.; Yuan, G.L.; Zhu, L.; Li, L.; Pei, C.; Hou, L.; Li, C., Jiang, X.Y.; Kong, X.H. Molecular characteristics of interleukin (IL)-17A/F3 and its immune response on the pathogen and functional regulation on cytokines in common carp *Cyprinus carpio* L. *Dev Comp Immunol.* **2023**, 139, 104566.
67. Livak, K.J.; Schmittgen, T.D. Analysis of relative gene expression data using real-time quantitative PCR and the $2^{-\triangle\triangle Ct}$ method. *Methods.* **2001**, 25, 402-408.
68. Bustin, S.A.; Benes, V.; Garson, J.A.; Hellemans, J.; Huggett, J.; Kubista, M.; Mueller, R.; Nolan, T.; Pfaffl, M.W.; Shipley, G.L.; Vandesompele, J.; Wittwer, C.T. The MIQE guidelines: minimum information for publication of quantitative real-time PCR experiments. *Clin. Chem.* **2015**, 55, 611-622.

International Journal of *Molecular Sciences*

2. Supplementary Tables

Table S1. Nucleotide sequences of qPCR primers used in the present study.

Gene	Accession number	Forward Primer (Sequence 5′ -3′)	Reverse Primer (Sequence 5′ -3′)
apoc2	XM_019075634.2	GAGAAGGGAACCATTGCCACT	CAGGTTCTTGGCTTTCTCATCC
bub1	XM_042777302.1	TACGACAGCCCACCAAACTC	GACACACCTTCCCCAACGAT
ccnb1	XM_019095451.2	GCGAAGAGTCGTGGTTATATCG	CAGACGGGTGTTCCTTGTGA
cdc20	XM_042718051.1	TTGGGGAAGGCAGAACTTGT	CAGATGTACTCGCTATCATCCTCC
chek2	XM_042723122.1	TGGTCAGTCCCAATCCCACAG	GCCCTGGCTTGATGATGTTGG
cpt1a	XM_042728180.1	TGGACCGGGCTCAAATCAAA	CTATGCCTGGGATGCGACTG
ef-1α	XM_042745044.1	CAAGGAAGTCAGCGCCTACA	CATCCCTTGAACCAGCCCAT
eif4e1b	XM_019098642.2	GCTGAGAACTGTGAGACGGT	TGCTCTTAGTTGCGGTGTCT
exo1	XM_042742200.1	AGCCTCTCTGTGGATGACCT	GTGCTTCCACACCCCTTCTT
gtse1	XM_042753016.1	GCTTCAGACCCCAGCAAAAA	CTGGGACCTTAAGTTTGGGGTT
kif11	XM_042742072.1	TGGAATTGCAGACTGGCAAAG	GGTAAATCTTGCCGCAGCTC
kif23	XM_042775284.1	TAGCCAGTCACCTCCAGACA	GTCCAGTGACCCGTTTCCTC
mcm4	XM_042752393.1	TCACAGACCCCAACTCCAGA	GTACCGGCTCTCCAACAACA
mrpl22	XM_042747680.1	CATGGACGTGGAATGTTCGG	GCCTGGTCGAATCCTGTCTT
nip7	XM_042775657.1	AGCTGGCTACAAATATCTCCCG	ATGGCGCAAGGAAATCGAGA
ppp1r3cb	XM_019089795.2	GATGTCACAGCAACTCTCCACA	TTCTTCAGCAACCGACTACGA
sod3	XM_042761153.1	GTGGTTTGCCTGCCGATGAT	CCGCCTGTGGATTGTAGTGA
wee2	XM_042744078.1	CTTGGCCTATGAGGTCCTGC	GAGGCTGTGCCAGTCATCTC

Table S2. Top 30 of GO analysis on DEGs of liver in the common carp after exposure to 0.45 and 1.35 μg /L FEN. Significance criteria: |log2 (Fold change)| ≥ 1 and adjusted $p \leq 0.05$ (corrected by Benjamini-Hochberg method).

Group	Category	Term	ID	p-value
0.45 μg/L	Biological process	positive regulation of acrosome reaction	GO:2000344	1.99×10^{-51}
		binding of sperm to zona pellucida	GO:0007339	1.95×10^{-38}
		egg coat formation	GO:0035803	3.86×10^{-36}
		cell division	GO:0051301	1.08×10^{-31}
		response to testosterone	GO:0033574	1.17×10^{-24}
		positive regulation of humoral immune response	GO:0002922	1.34×10^{-22}
		response to progesterone	GO:0032570	6.44×10^{-22}
		acrosomal vesicle exocytosis	GO:0060478	9.91×10^{-20}
		DNA replication initiation	GO:0006270	1.43×10^{-17}
		negative regulation of binding of sperm to zona pellucida	GO:2000360	4.99×10^{-14}
	Cellular compo-	MCM complex	GO:0042555	1.69×10^{-14}

	nent	cell	GO:0005623	7.27×10^{-14}
		condensed chromosome kinetochore	GO:0000777	1.03×10^{-13}
		apical part of cell	GO:0045177	1.90×10^{-10}
		nucleus	GO:0005634	4.62×10^{-10}
		HAUS complex	GO:0070652	6.94×10^{-9}
		P granule	GO:0043186	2.53×10^{-8}
		nuclear origin of replication recognition complex	GO:0005664	4.80×10^{-8}
		cyclin-dependent protein kinase holoenzyme complex	GO:0000307	5.20×10^{-8}
		chromatoid body	GO:0033391	7.06×10^{-8}
	Molecular function	acrosin binding	GO:0032190	3.22×10^{-59}
		cysteine-type endopeptidase inhibitor activity	GO:0004869	5.24×10^{-9}
		DNA helicase activity	GO:0003678	9.11×10^{-9}
		DNA replication origin binding	GO:0003688	1.14×10^{-8}
		ribonucleoside-diphosphate reductase activity, thioredoxin disulfide as acceptor	GO:0004748	2.68×10^{-6}
		superoxide dismutase activity	GO:0004784	1.14×10^{-5}
		single-stranded DNA binding	GO:0003697	2.80×10^{-5}
		peptidase inhibitor activity	GO:0030414	3.34×10^{-5}
		cyclin-dependent protein serine/threonine kinase regulator activity	GO:0016538	8.49×10^{-5}
		superoxide dismutase copper chaperone activity	GO:0016532	9.35×10^{-5}
1.35 μg/L	Biological process	positive regulation of acrosome reaction	GO:2000344	8.05×10^{-53}
		binding of sperm to zona pellucida	GO:0007339	7.49×10^{-38}
		egg coat formation	GO:0035803	1.09×10^{-35}
		cell division	GO:0051301	1.35×10^{-31}
		response to testosterone	GO:0033574	3.21×10^{-24}
		positive regulation of humoral immune response	GO:0002922	2.44×10^{-22}
		response to progesterone	GO:0032570	1.80×10^{-21}
		acrosomal vesicle exocytosis	GO:0060478	1.80×10^{-19}
		DNA replication initiation	GO:0006270	9.53×10^{-19}
		DNA replication	GO:0006260	1.88×10^{-15}
	Cellular component	MCM complex	GO:0042555	3.90×10^{-16}
		cell	GO:0005623	1.07×10^{-15}
		nucleus	GO:0005634	2.55×10^{-13}
		condensed chromosome kinetochore	GO:0000777	9.99×10^{-12}
		apical part of cell	GO:0045177	4.42×10^{-10}

		P granule	GO:0043186	1.72×10^{-9}
		chromatin	GO:0000785	2.31×10^{-9}
		nuclear MIS12/MIND complex	GO:0000818	2.31×10^{-9}
		pi-body	GO:0071546	2.83×10^{-9}
		HAUS complex	GO:0070652	9.13×10^{-9}
	Molecular function	acrosin binding	GO:0032190	1.75×10^{-58}
		cysteine-type endopeptidase inhibitor activity	GO:0004869	4.35×10^{-11}
		DNA replication origin binding	GO:0003688	4.51×10^{-10}
		DNA helicase activity	GO:0003678	2.59×10^{-7}
		cyclin-dependent protein serine/threonine kinase inhibitor activity	GO:0004861	3.18×10^{-6}
		ribonucleoside-diphosphate reductase activity, thioredoxin disulfide as acceptor	GO:0004748	3.25×10^{-6}
		DNA clamp loader activity	GO:0003689	1.38×10^{-5}
		superoxide dismutase activity	GO:0004784	1.38×10^{-5}
		peptidase inhibitor activity	GO:0030414	4.17×10^{-5}
		single-stranded DNA binding	GO:0003697	4.30×10^{-5}

Table S3. Top 30 of GO analysis on DEGs of kidney in the common carp after exposure to 0.45 and 1.35 μg/L FEN. Significance criteria: |log2 (Fold change)| ≥ 1 and adjusted $p \leq 0.05$ (corrected by Benjamini-Hochberg method).

Group	Category	Term	ID	p-value
0.45 μg/L	Biological process	positive regulation of acrosome reaction	GO:2000344	1.93×10^{-51}
		binding of sperm to zona pellucida	GO:0007339	4.39×10^{-44}
		egg coat formation	GO:0035803	2.31×10^{-37}
		response to testosterone	GO:0033574	7.92×10^{-25}
		response to progesterone	GO:0032570	7.23×10^{-22}
		positive regulation of humoral immune response	GO:0002922	7.56×10^{-22}
		acrosomal vesicle exocytosis	GO:0060478	5.52×10^{-19}
		negative regulation of binding of sperm to zona pellucida	GO:2000360	2.88×10^{-13}
		piRNA metabolic process	GO:0034587	7.32×10^{-12}
		positive regulation of T cell proliferation	GO:0042102	9.78×10^{-11}
	Cellular component	cell	GO:0005623	3.22×10^{-21}
		apical part of cell	GO:0045177	3.08×10^{-12}
		P granule	GO:0043186	1.56×10^{-10}
		pi-body	GO:0071546	4.76×10^{-9}
		extracellular region	GO:0005576	5.31×10^{-8}
		extracellular space	GO:0005615	1.51×10^{-7}
		chromatoid body	GO:0033391	1.71×10^{-7}
		laminin-1 complex	GO:0005606	1.95×10^{-6}

		MCM complex	GO:0042555	1.95×10^{-6}
		cytoplasmic exosome (RNase complex)	GO:0000177	4.72×10^{-6}
	Molecular function	acrosin binding	GO:0032190	7.86×10^{-60}
		cysteine-type endopeptidase inhibitor activity	GO:0004869	8.17×10^{-20}
		serine-type endopeptidase inhibitor activity	GO:0004867	1.16×10^{-8}
		superoxide dismutase activity	GO:0004784	6.65×10^{-7}
		endopeptidase inhibitor activity	GO:0004866	1.47×10^{-6}
		superoxide dismutase copper chaperone activity	GO:0016532	4.70×10^{-6}
		peptidase inhibitor activity	GO:0030414	4.72×10^{-6}
		glycosphingolipid binding	GO:0043208	4.72×10^{-6}
		DNA topoisomerase type I activity	GO:0003917	1.15×10^{-5}
		helicase activity	GO:0004386	3.12×10^{-5}
1.35 μg/L	Biological process	positive regulation of acrosome reaction	GO:2000344	4.61×10^{-52}
		binding of sperm to zona pellucida	GO:0007339	7.43×10^{-41}
		egg coat formation	GO:0035803	9.20×10^{-38}
		response to testosterone	GO:0033574	1.08×10^{-26}
		response to progesterone	GO:0032570	1.56×10^{-23}
		positive regulation of humoral immune response	GO:0002922	4.54×10^{-22}
		acrosomal vesicle exocytosis	GO:0060478	3.33×10^{-19}
		piRNA metabolic process	GO:0034587	7.24×10^{-16}
		negative regulation of binding of sperm to zona pellucida	GO:2000360	1.72×10^{-13}
		DNA methylation involved in gamete generation	GO:0043046	5.63×10^{-11}
	Cellular component	cell	GO:0005623	1.12×10^{-22}
		apical part of cell	GO:0045177	2.25×10^{-13}
		pi-body	GO:0071546	3.68×10^{-11}
		extracellular space	GO:0005615	5.89×10^{-10}
		P granule	GO:0043186	2.56×10^{-9}
		extracellular region	GO:0005576	7.30×10^{-8}
		chromatoid body	GO:0033391	1.32×10^{-7}
		MCM complex	GO:0042555	1.56×10^{-6}
		endosome lumen	GO:0031904	7.58×10^{-6}
		extracellular matrix	GO:0031012	2.71×10^{-5}
	Molecular function	acrosin binding	GO:0032190	1.80×10^{-60}
		cysteine-type endopeptidase inhibitor activity	GO:0004869	7.01×10^{-16}
		steroid hormone receptor binding	GO:0035258	8.05×10^{-8}

		endopeptidase inhibitor activity	GO:0004866	9.02×10^{-8}
		superoxide dismutase activity	GO:0004784	5.45×10^{-7}
		serine-type endopeptidase inhibitor activity	GO:0004867	7.09×10^{-7}
		peptidase inhibitor activity	GO:0030414	3.77×10^{-6}
		superoxide dismutase copper chaperone activity	GO:0016532	3.98×10^{-6}
		hemoglobin binding	GO:0030492	4.04×10^{-6}
		bile acid transmembrane transporter activity	GO:0015125	1.71×10^{-5}

Table S4. Significantly enriched KEGG pathways of DEGs in carp liver in FEN exposure groups.

Group	KEGG pathway	Second-level KEGG pathway	The top level KEGG pathway	Pathway ID	p-value
0.45 μg/L	Cell cycle	Cell growth and death	Cellular Processes	path:ccar04110	2.20×10^{-27}
	p53 signaling pathway			path:ccar04115	9.23×10^{-6}
	Oocyte meiosis			path:ccar04114	5.34×10^{-5}
	DNA replication	Replication and repair	Genetic Information Processing	path:ccar03030	5.77×10^{-27}
	Mismatch repair			path:ccar03430	2.62×10^{-18}
	Fanconi anemia pathway			path:ccar03460	4.58×10^{-9}
	Nucleotide excision repair			path:ccar03420	5.10×10^{-9}
	Homologous recombination			path:ccar03440	8.39×10^{-6}
	Base excision repair			path:ccar03410	5.80×10^{-4}
	Sulfur relay system	Folding, sorting and degradation		path:ccar04122	3.97×10^{-2}
	RNA degradation			path:ccar03018	1.64×10^{-4}
	RNA transport	Translation		path:ccar03013	3.41×10^{-6}
	Ribosome biogenesis in eukaryotes			path:ccar03008	1.06×10^{-3}
	mRNA surveillance pathway			path:ccar03015	2.22×10^{-2}
	RNA polymerase	Transcription		path:ccar03020	5.93×10^{-3}
	Basal transcription factors			path:ccar03022	1.12×10^{-2}
	Biosynthesis of unsaturated fatty acids	Lipid metabolism	Metabolism	path:ccar01040	1.93×10^{-2}
	Fatty acid elongation			path:ccar00062	2.58×10^{-2}
	Pyrimidine metabolism	Nucleotide metabolism		path:ccar00240	6.10×10^{-5}
	Purine metabolism			path:ccar00230	4.69×10^{-3}
	Drug metabolism - other enzymes	Xenobiotics biodegradation and metabolism		path:ccar00983	4.36×10^{-3}
	Glutathione metabolism	Metabolism of other amino acids		path:ccar00480	3.13×10^{-2}

	Arginine biosynthesis	Amino acid metabolism		path:ccar00220	4.35×10^{-2}
	Progesterone-mediated oocyte maturation	Endocrine system	Organismal Systems	path:ccar04914	7.94×10^{-5}
	Cytosolic DNA-sensing pathway	Immune system		path:ccar04623	2.53×10^{-3}
1.35 μg/L	Cell cycle	Cell growth and death	Cellular Processes	path:ccar04110	1.84×10^{-36}
	Oocyte meiosis			path:ccar04114	8.82×10^{-7}
	p53 signaling pathway			path:ccar04115	2.96×10^{-6}
	Cellular senescence			path:ccar04218	1.21×10^{-2}
	DNA replication	Replication and repair	Genetic Information Processing	path:ccar03030	3.80×10^{-34}
	Mismatch repair			path:ccar03430	1.17×10^{-19}
	Fanconi anemia pathway			path:ccar03460	5.94×10^{-12}
	Nucleotide excision repair			path:ccar03420	8.99×10^{-11}
	Homologous recombination			path:ccar03440	2.46×10^{-8}
	Base excision repair			path:ccar03410	1.91×10^{-5}
	Sulfur relay system	Folding, sorting and degradation		path:ccar04122	3.51×10^{-3}
	RNA degradation			path:ccar03018	4.95×10^{-3}
	RNA transport	Translation		path:ccar03013	5.06×10^{-8}
	mRNA surveillance pathway			path:ccar03015	1.14×10^{-2}
	Spliceosome	Transcription		path:ccar03040	3.07×10^{-2}
	Biosynthesis of unsaturated fatty acids	Lipid metabolism	Metabolism	path:ccar01040	2.08×10^{-2}
	Fatty acid elongation			path:ccar00062	2.77×10^{-2}
	Pyrimidine metabolism	Nucleotide metabolism		path:ccar00240	6.63×10^{-7}
	Purine metabolism			path:ccar00230	1.17×10^{-2}
	Drug metabolism - other enzymes	Xenobiotics biodegradation and metabolism		path:ccar00983	1.63×10^{-3}
	Glutathione metabolism	Metabolism of other amino acids		path:ccar00480	3.40×10^{-2}
	Arginine biosynthesis	Amino acid metabolism		path:ccar00220	4.57×10^{-2}
	Progesterone-mediated oocyte maturation	Endocrine system	Organismal Systems	path:ccar04914	3.03×10^{-6}

Table S5. Significantly enriched KEGG pathways of DEGs in carp kidney in FEN exposure groups.

Group	KEGG pathway	Second-level KEGG pathway	The top level KEGG pathway	Pathway ID	p-value
0.45 μg/L	Cell cycle	Cell growth and death	Cellular Processes	path:ccar04110	5.20×10^{-13}
	Oocyte meiosis			path:ccar04114	4.00×10^{-3}

	p53 signaling pathway			path:ccar04115	5.39×10^{-3}
	Phagosome	Transport and catabolism		path:ccar04145	2.15×10^{-2}
	DNA replication	Replication and repair	Genetic Information Processing	path:ccar03030	5.56×10^{-7}
	Fanconi anemia pathway			path:ccar03460	5.56×10^{-7}
	Nucleotide excision repair			path:ccar03420	1.75×10^{-3}
	Homologous recombination			path:ccar03440	1.88×10^{-3}
	Mismatch repair			path:ccar03430	2.53×10^{-3}
	RNA degradation	Folding, sorting and degradation		path:ccar03018	2.71×10^{-5}
	RNA transport	Translation		path:ccar03013	3.40×10^{-6}
	mRNA surveillance pathway			path:ccar03015	1.08×10^{-3}
	RNA polymerase	Transcription		path:ccar03020	1.65×10^{-3}
	Basal transcription factors			path:ccar03022	1.46×10^{-2}
	Fatty acid elongation	Lipid metabolism	Metabolism	path:ccar00062	2.58×10^{-3}
	Biosynthesis of unsaturated fatty acids			path:ccar01040	6.89×10^{-3}
	Ubiquinone and other terpenoid-quinone biosynthesis	Metabolism of cofactors and vitamins		path:ccar00130	1.47×10^{-2}
	Cytosolic DNA-sensing pathway	Immune system	Organismal Systems	path:ccar04623	2.39×10^{-4}
	PPAR signaling pathway	Endocrine system		path:ccar03320	1.70×10^{-2}
1.35 μg/L	Cell cycle	Cell growth and death	Cellular Processes	path:ccar04110	1.35×10^{-8}
	p53 signaling pathway			path:ccar04115	3.67×10^{-3}
	Ferroptosis			path:ccar04216	2.63×10^{-2}
	Phagosome	Transport and catabolism		path:ccar04145	4.62×10^{-2}
	DNA replication	Replication and repair	Genetic Information Processing	path:ccar03030	4.27×10^{-4}
	Fanconi anemia pathway			path:ccar03460	7.71×10^{-3}
	Homologous recombination			path:ccar03440	2.15×10^{-2}
	Mismatch repair			path:ccar03430	4.44×10^{-2}
	RNA degradation	Folding, sorting and degradation		path:ccar03018	1.67×10^{-3}
	RNA transport	Translation		path:ccar03013	1.12×10^{-5}
	mRNA surveillance pathway			path:ccar03015	1.75×10^{-3}
	RNA polymerase	Transcription		path:ccar03020	2.52×10^{-2}
	Basal transcription factors			path:ccar03022	3.70×10^{-2}
	Biosynthesis of unsaturated fatty acids	Lipid metabolism	Metabolism	path:ccar01040	1.26×10^{-3}
	Fatty acid elongation			path:ccar00062	1.94×10^{-3}
	Steroid hormone biosynthe-			path:ccar00140	2.18×10^{-2}

	sis				
	Glycerolipid metabolism			path:ccar00561	3.25×10^{-2}
	Taurine and hypotaurine metabolism	Metabolism of other amino acids		path:ccar00430	1.82×10^{-3}
	Glutathione metabolism			path:ccar00480	3.24×10^{-2}
	Retinol metabolism	Metabolism of cofactors and vitamins		path:ccar00830	1.56×10^{-2}
	Starch and sucrose metabolism	Carbohydrate metabolism		path:ccar00500	4.30×10^{-2}
	Cytosolic DNA-sensing pathway	Immune system	Organismal Systems	path:ccar04623	2.65×10^{-3}
	PPAR signaling pathway	Endocrine system		path:ccar03320	2.84×10^{-2}
	Hedgehog signaling pathway	Signal transduction	Environmental Information Processing	path:ccar04340	2.09×10^{-2}

3. Supplementary Figures

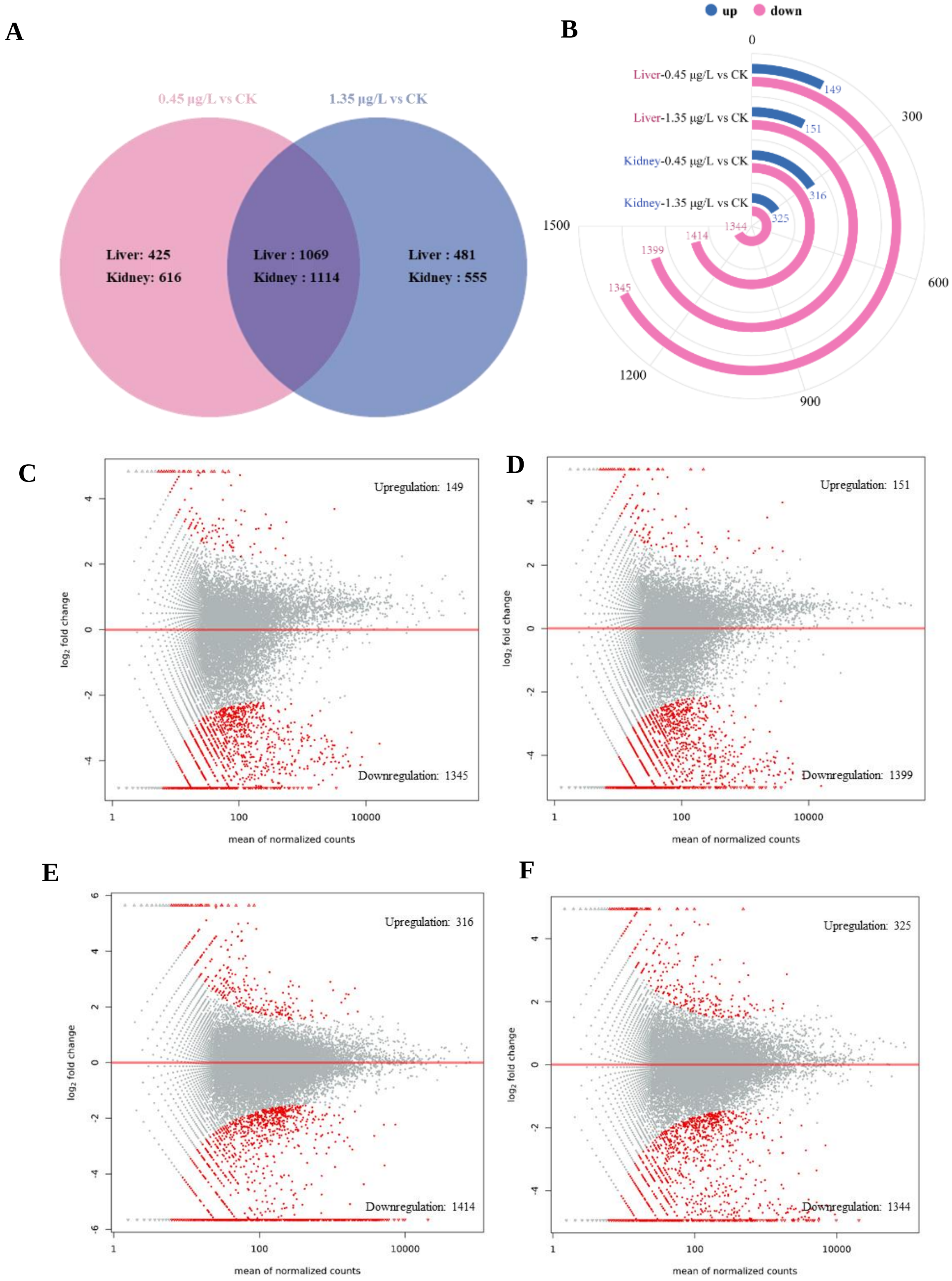

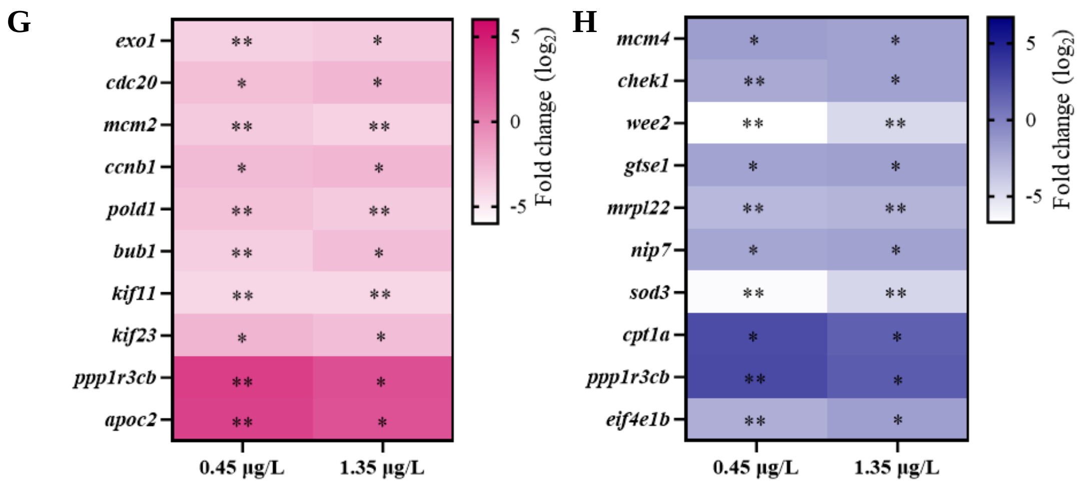

Figure S1. Transcriptional alterations of carp liver and kidney tissues after FEN treatment. (A) Venn diagram of DEGs in fish liver and kidney. (B) DEGs in different comparisons in the liver and kidney. (C) Volcano plots of DEGs in the liver of fish after 0.45 μg/L FEN exposure. (D) Volcano plots of DEGs in the liver of fish after 1.35 μg/L FEN exposure. (E) Volcano plots of DEGs in the kidney after 0.45 μg/L FEN exposure. (F) Volcano plots of DEGs in the kidney after 1.35 μg/L FEN exposure. (G) Gene expression of RNA-Seq in the liver after FEN exposure. (H) Gene expression of RNA-Seq in the kidney after FEN exposure. * $p < 0.05$ and ** $p < 0.01$ compared with the controls.

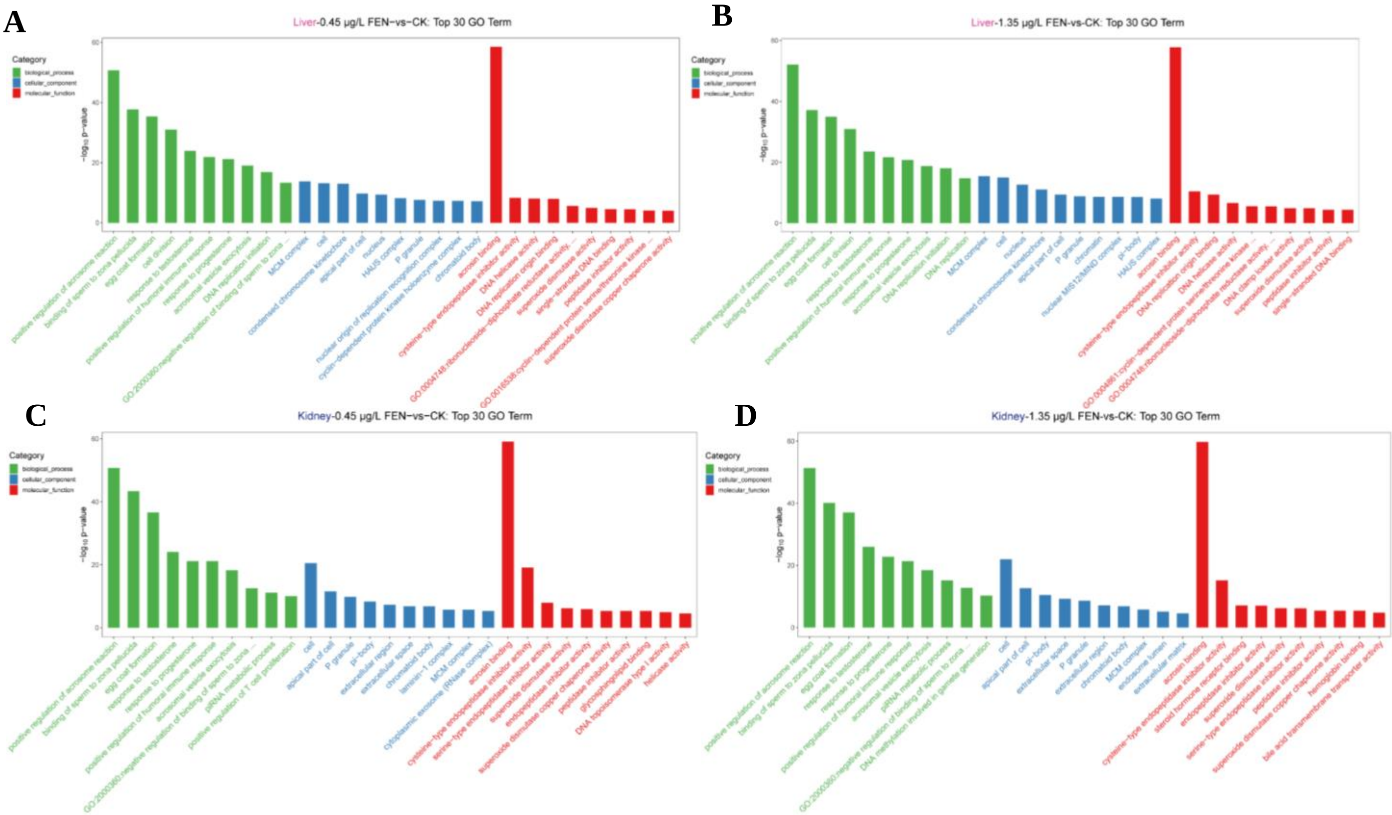

Figure S2. GO classification analysis. (A) Top 30 GO terms on DEGs of liver in carp after exposure to 0.45 μg/L FEN. (B) Top 30 GO terms on DEGs of liver in carp after exposure to 1.35 μg/L FEN. (C) Top 30 GO terms on DEGs of kidney in carp after exposure to 0.45 μg/L FEN. (D) Top 30 of GO analysis on DEGs of kidney in carp after exposure to 1.35 μg/L FEN.

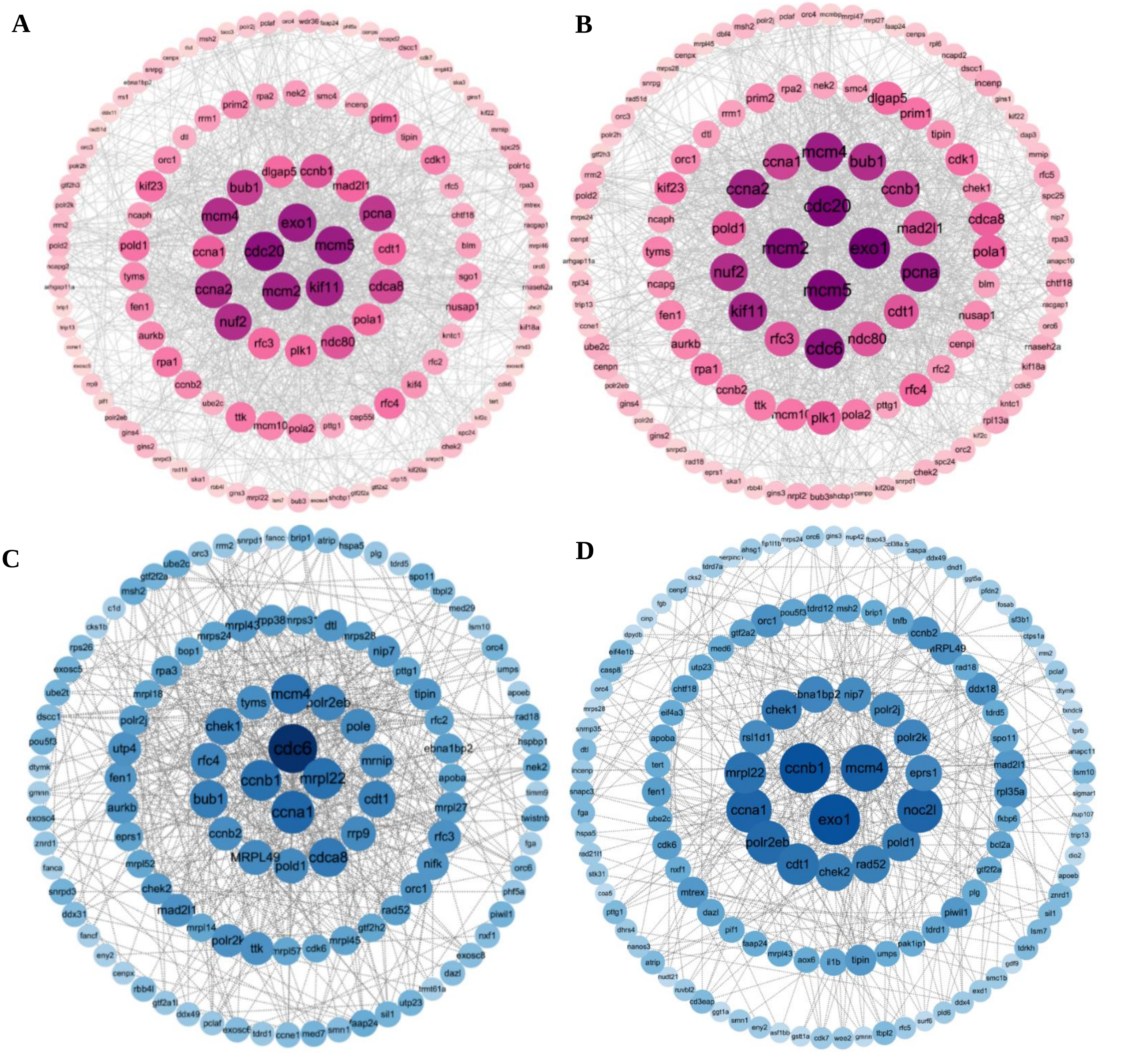

Figure S3. PPI network analysis of DEGs in the liver and kidney of common carp after FEN exposure. (A) The hub genes in carp liver were classified by PPI network research after 0.45 μg/L FEN exposure. (B) The hub genes in carp liver were classified by PPI network research after 1.35 μg/L FEN exposure. (C) The hub genes in carp kidney were classified by PPI network research after 0.45 μg/L FEN exposure. (D) The hub genes in carp kidney were classified by PPI network research after 1.35 μg/L FEN exposure.

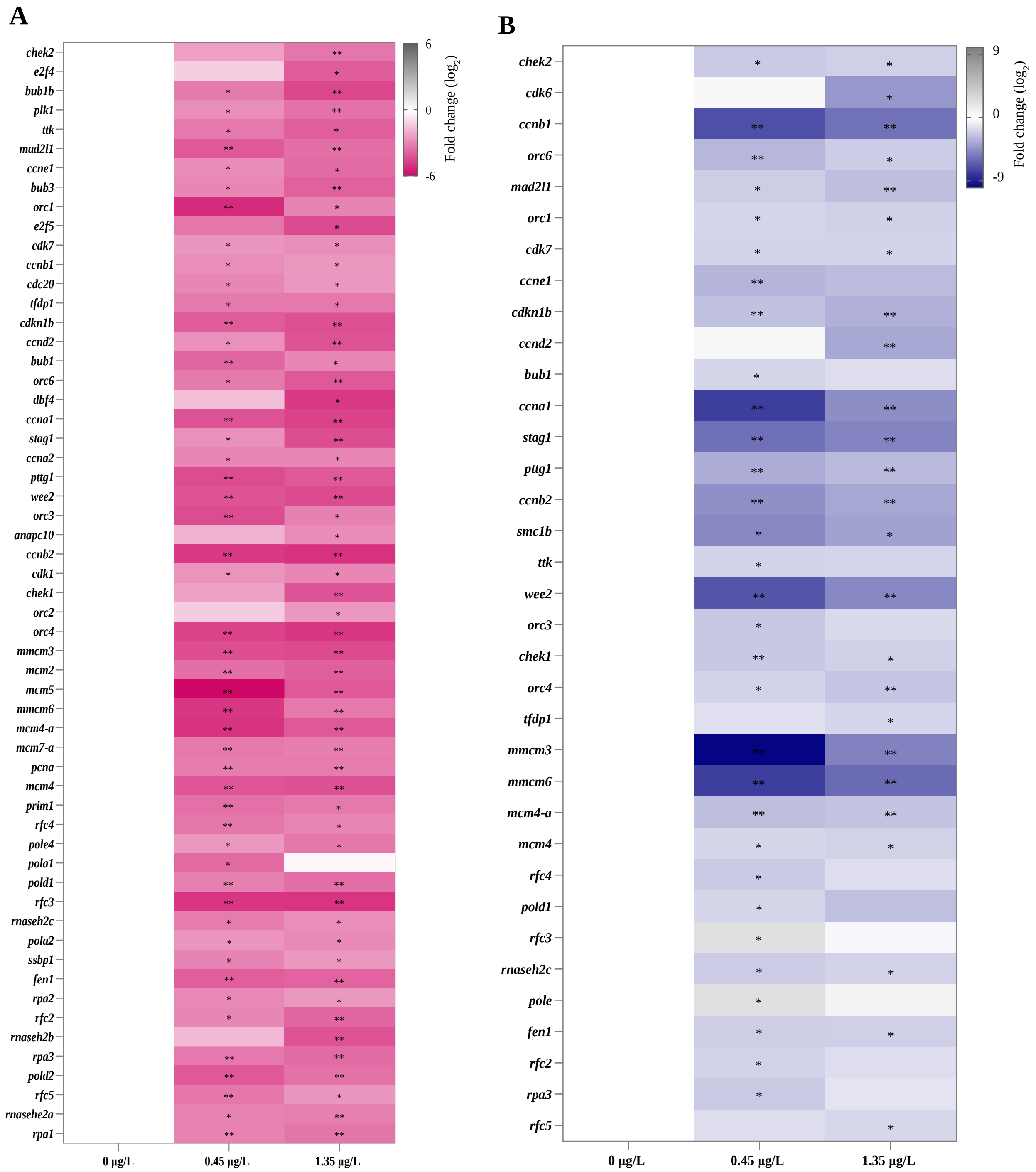

Figure S4. Summary of expression levels of DEGs related to cell cycle and DNA replication in the carp (A) liver and (B) kidney following FEN stress. * $p < 0.05$ and ** $p < 0.01$ compared with the controls.
